# Detailed analysis of family with autosomal recessive bestrophinopathy associated with new *BEST1* mutation

**DOI:** 10.1007/s10633-016-9540-3

**Published:** 2016-04-12

**Authors:** Daiki Kubota, Kiyoko Gocho, Keiichiro Akeo, Sachiko Kikuchi, Michitaka Sugahara, Celso Soiti Matsumoto, Kei Shinoda, Atsushi Mizota, Kunihiko Yamaki, Hiroshi Takahashi, Shuhei Kameya

**Affiliations:** Department of Ophthalmology, Nippon Medical School Chiba Hokusoh Hospital, 1715 Kamagari, Inzai, Chiba 270-1694 Japan; Inoue Eye Clinic, 4-3 Surugadai, Kanda, Chiyoda-ku, Tokyo, 101-0062 Japan; Sugahara Eye Clinic, 1-13-3, Minami-senju, Arakawa-ku, Tokyo, 116-0003 Japan; Department of Ophthalmology, Teikyo University School of Medicine, 2-11-1 Kaga, Itabashi-ku, Tokyo, 173-8605 Japan; Department of Ophthalmology, Nippon Medical School, 1-1-5 Sendagi, Bunkyo-ku, Tokyo, 113-8602 Japan

**Keywords:** Autosomal recessive bestrophinopathy, BEST1, Fundus autofluorescence, Electro-oculography (EOG)

## Abstract

**Purpose:**

To describe the clinical and genetic findings in a patient with autosomal recessive bestrophinopathy (ARB) and his healthy parents.

**Methods:**

The patient and his healthy non-consanguineous parents underwent detailed ophthalmic evaluations including electro-oculography (EOG), spectral-domain optical coherence tomography (SD-OCT), and fundus autofluorescence (FAF) imaging. Mutation analysis of the *BEST1* gene was performed by Sanger sequencing.

**Results:**

The FAF images showed multiple spots of increased autofluorescence, and the sites of these spots corresponded to the yellowish deposits detected by ophthalmoscopy. SD-OCT showed cystoid macular changes and a shallow serous macular detachment. The Arden ratio of the EOG was markedly reduced to 1.1 in both eyes. Genetic analysis of the proband detected two sequence variants of the *BEST1* gene in the heterozygous state: a novel variant c.717delG, p.V239VfsX2 and an already described c.763C>T, p.R255W variant associated with Best vitelliform macular dystrophy and ARB. The proband’s father carried the c.717delG, p.V239VfsX2 variant in the heterozygous state, and the mother carried the c.763C>T, p.R255W variant in the heterozygous state. The parents who were heterozygous for the *BEST1* variants had normal visual acuity, EOG, SD-OCT, and FAF images.

**Conclusions:**

In a truncating *BEST1* mutation, the phenotype associated with ARB is most likely due to a marked decrease in the expression of *BEST1* promoted by the nonsense-mediated decay surveillance mechanism, and it may depend on the position of the premature termination of the codon created.

## Introduction

*BEST1* (VMD2) is a gene located on chromosome 11 (11q12.3) that encodes for the 585 amino acid transmembrane protein bestrophin 1 which is located on the basolateral aspect of retinal pigment epithelial (RPE) cells [1, 2]. Although the functional role of bestrophin-1 within the RPE has not been determined definitively, it has been postulated to function as a Ca^2+^-activated Cl^−^ channel [3], a regulator of voltage-gated Ca^2+^ channels [4], and a HCO_3_^−^ channel [5]. Mutations in *BEST1* therefore affect the RPE metabolism and consequently the outer retinal function with which the RPE is intimately associated.

Mutations of the *BEST1* gene have been associated with different ocular phenotypes [6]. The first disease shown to be caused by *BEST1* sequence variants was Best vitelliform macular dystrophy (BVMD) [1], a retinal disease characterized by bilateral yellowish yolk-colored lesion in the macula. *BEST1* mutations are also associated with several other eye diseases including adult-onset vitelliform macular dystrophy (AOVMD) [7], autosomal dominant vitreo-retinochoroidopathy (ADVIRC) [8], retinitis pigmentosa [9], and the microcornea, retinal dystrophy, cataract, and posterior staphyloma (MRCS) syndrome [10].

Autosomal recessive bestrophinopathy (ARB), first described in detail in 2008, is another member of the phenotypic spectrum associated with mutations in the *BEST1* gene [11]. The characteristics of this disorder include a progressive reduction in central vision, absence of the electro-oculographic (EOG) light rise, and reduced full-field electroretinograms (ERGs). None of the patients have the vitelliform lesions typical of Best disease, but have a diffuse irregularity of the reflex from the RPE including dispersed punctate flecks [11]. All of the patients have an accumulation of fluid within and/or beneath the neurosensory retina in the macular area [11].

ARB has been reported to be due to either compound heterozygous or homozygous *BEST1* gene mutations [6, 11]. A recent manuscript described an ocular phenotype similar to ARB associated with a single heterozygous mutation of the *BEST1* gene [12].

Several mutations associated with ARB have been reported to be involved in causing dominant Best disease when they were present in the heterozygous state [6, 7, 13–15]. The clinical phenotype of some patients with recessive bestrophinopathy is distinct from that seen in Best disease, while in others it is similar to the typical phenotype observed in autosomal dominant vitelliform dystrophy [16, 17]. Identification of additional families with recessive bestrophinopathy and detailed characterization of the clinical phenotypes of homozygous and heterozygous individuals will assist in establishing the phenotype–genotype correlations in patients with *BEST1*-associated diseases.

## Methods

The protocol of this study conformed to the tenets of the Declaration of Helsinki and was approved by the Institutional Review Board of the Nippon Medical School. A signed written informed consent was obtained from the patient and his parents after the nature and possible consequences of the study were explained.

Blood samples were collected from the patient and his parents, and genomic DNA was isolated from the peripheral white blood cells using a blood DNA isolation kit (NucleoSpin Blood XL; Macherey–Nagel, Germany). The DNA was used as the template to amplify the *BEST1* gene. The coding regions and flanking introns of the *BEST1* gene were amplified by polymerase chain reaction (PCR) using primers synthesized by Greiner Bio-One (Tokyo, Japan). The PCR products were purified (ExoSAP-IT; USB Corp., USA) and were used as the template for sequencing. Both strands were sequenced on an automated sequencer (Bio Matrix Research; Chiba, Japan).

The ophthalmological examinations included measurements of the best-corrected visual acuity (BCVA), refractive error and axial length, slit-lamp biomicroscopy, ophthalmoscopy, fundus photography, fundus autofluorescence (FAF) imaging, fluorescein angiography (FA), SD-OCT, full-field electroretinography (ERG), multifocal ERGs (mfERGs), and electro-oculography (EOG). The EOGs and ERGs were recorded using an extended testing protocol conforming to the International Society for Clinical Electrophysiology of Vision standards. The ERGs were elicited and recorded with a LED built-in electrode (LE2000, Tomey, Japan). The mfERGs were recorded using a commercial mfERG system (VERIS Science; Electro-Diagnostic Imaging, Inc. Redwood City, CA, USA). The FAF images were acquired with the TRC-NW8Fplus retinal camera (TOPCON, Tokyo, Japan), and the SD-OCT images were acquired with a Cirrus HD-OCT (Carl Zeiss Meditec).

## Results

**Fig. 1:**
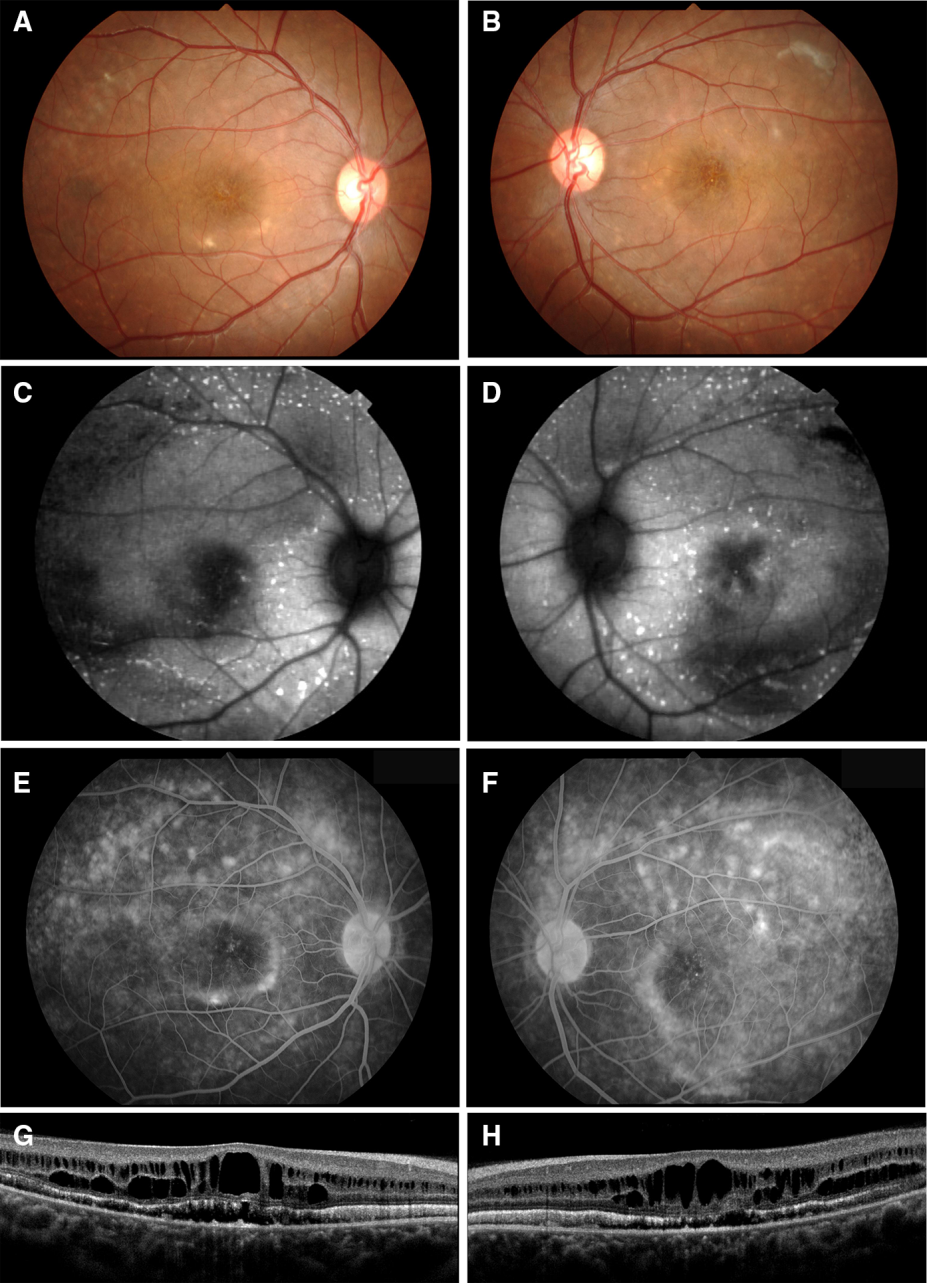
Fundus photographs, autofluorescence images, fluorescein angiograms, and SD-OCT images from patient with autosomal recessive bestrophinopathy (ARB) (proband, II-1). Fundus photographs (**a**, **b**), autofluorescence images (**c**, **d**), fluorescein angiograms (**e**, **f**), and SD-OCT images (**g**, **h**) are shown. Results from the right eye (**a**, **c**, **e**, **g**) and left eye (**b**, **d**, **f**, **h**) are shown. Fundus photograph shows cystoid macular lesions and multiple yellowish deposits throughout the posterior pole of both eyes. FAF images show multiple hyper-autofluorescent regions in the peripheral retina of both eyes. FAF images also show a hypo-autofluorescent lesion in the macular of both eyes. Fluorescein angiograms show widespread patchy hyper-fluorescence. The SD-OCT images show cystoid macular changes and shallow serous retinal detachments in both eyes. There is also a thickening and hyper-reflectivity at the areas corresponding to the ellipsoid and interdigitation zones

The patient was a 25-year-old man whose decimal best-corrected visual acuity (BCVA) was 0.9 in the right eye and 0.3 in the left eye. His refraction was S + 0.5 C-1.25 at 180 in the right eye and S + 0.5 C-2.0 at 175 in the left eye. Axial length was 23.71 mm in the right eye and 23.85 mm in the left eye. The intraocular pressure and anterior ocular segments were within normal limits in both eyes. Fundus examinations revealed a cystoid macular lesion and multiple yellowish deposits throughout the posterior pole of both eyes (Fig. 1). The vitelliform lesions that are typical of Best disease were not observed (Fig. 1). FAF imaging showed multiple hyper-autofluorescent spots in the peripheral retina of both eyes, and the site of the spots corresponded with the yellowish deposits observed by ophthalmoscopy (Fig. 1). FAF imaging also detected a hypo-autofluorescent lesion in the macula of both eyes (Fig. 1). FA showed widespread patchy hyper-fluorescence (Fig. 1). The SD-OCT images showed cystoid changes in the macula and shallow serous retinal detachments in both eyes. There was a thickening and hyper-reflectivity at the areas corresponding to ellipsoid and interdigitation zones of the photoreceptors in the SD-OCT images (Fig. 1).

**Fig. 2:**
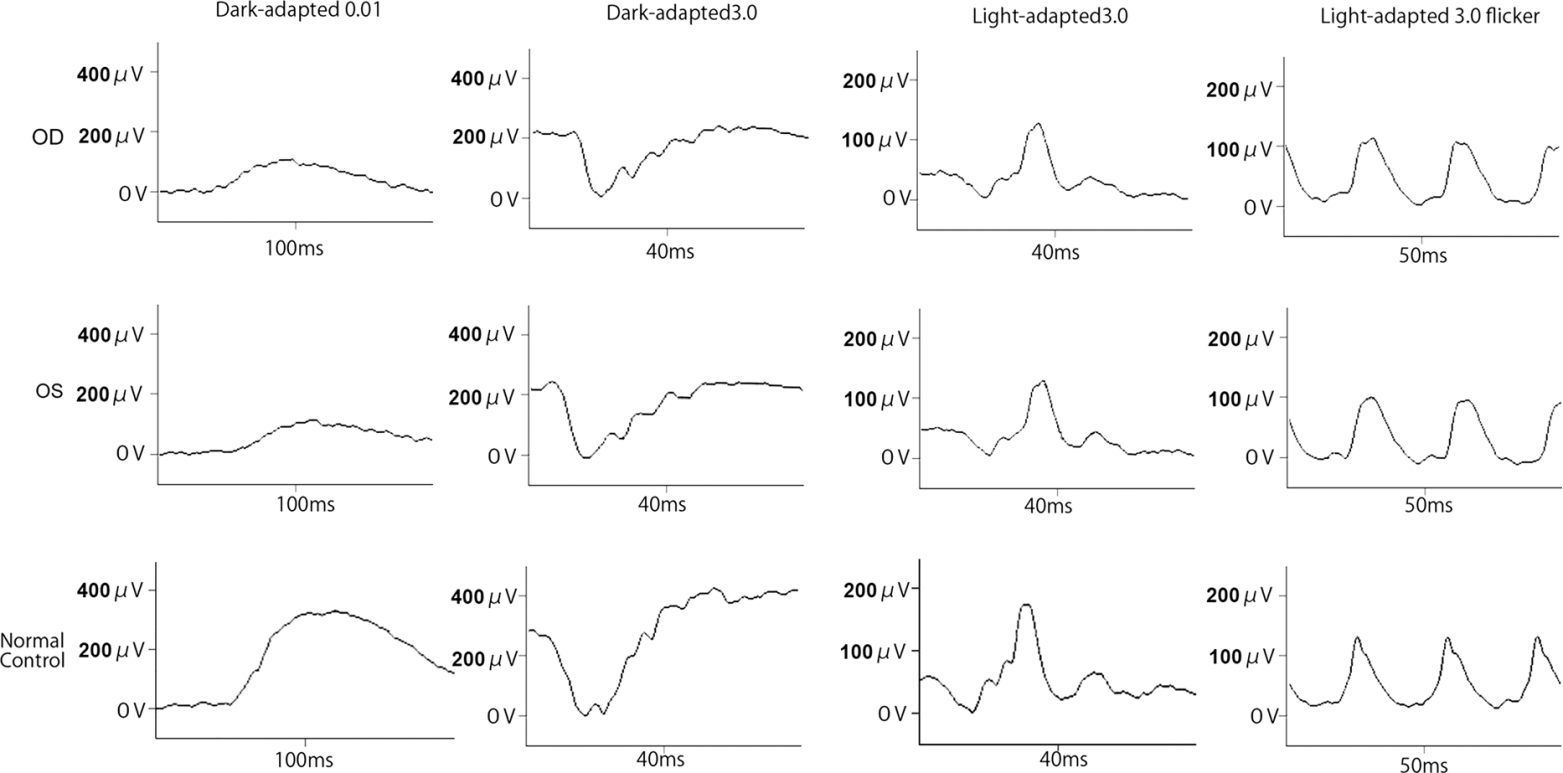
Full-field electroretinograms (ERGs). Full-field ERGs recorded from the right eye (*top*) and left eye (*middle*) of the proband (II-1) are shown. The ERGs recorded from a normal control are also shown (*bottom*). The dark-adapted 0.01, dark-adapted 3.0, light-adapted 3.0, and light-adapted 3.0 flicker ERGs are shown. The results of all the responses show a slight reduction of the b-wave amplitudes in both eyes

**Fig. 3:**
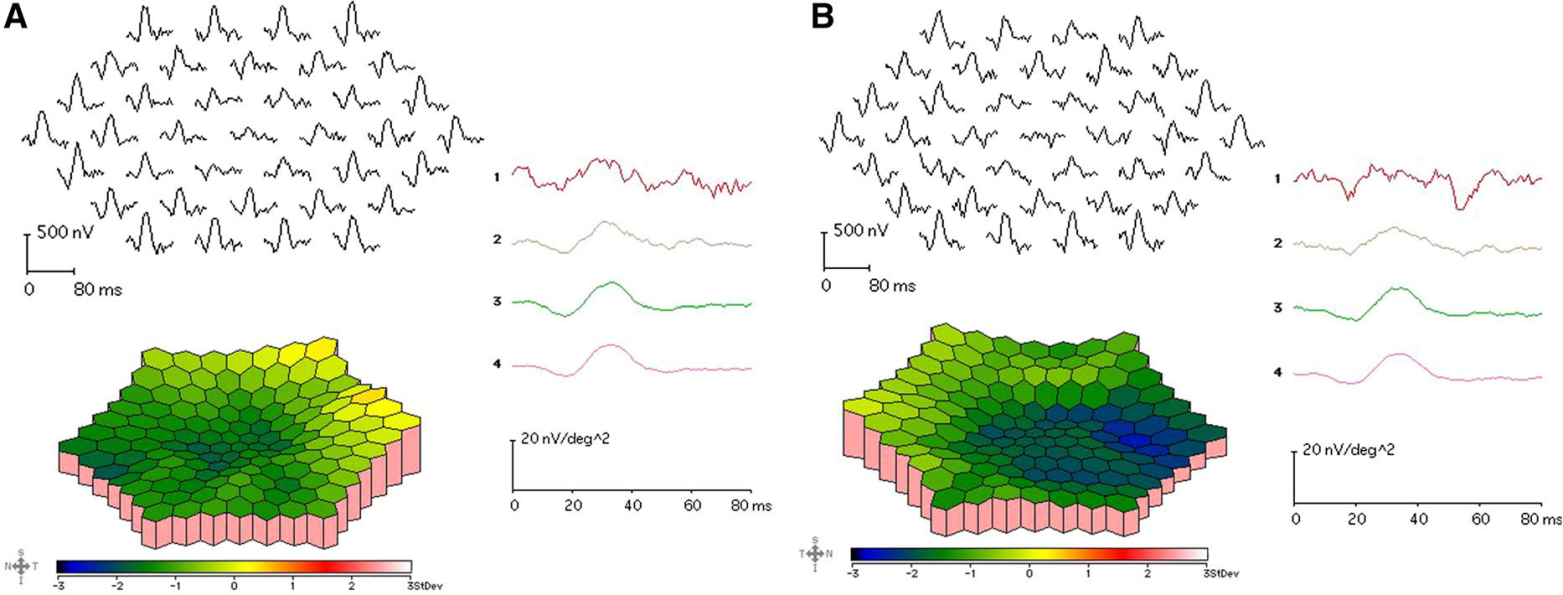
Multifocal ERGs. The mfERGs, topographic map, and average densities of the rings of the multifocal ERGs of right eye (**a**) and left eye (**b**) of the proband are shown. The amplitudes of the mfERGs in the foveal area are severely reduced in both eyes

**Fig. 4:**
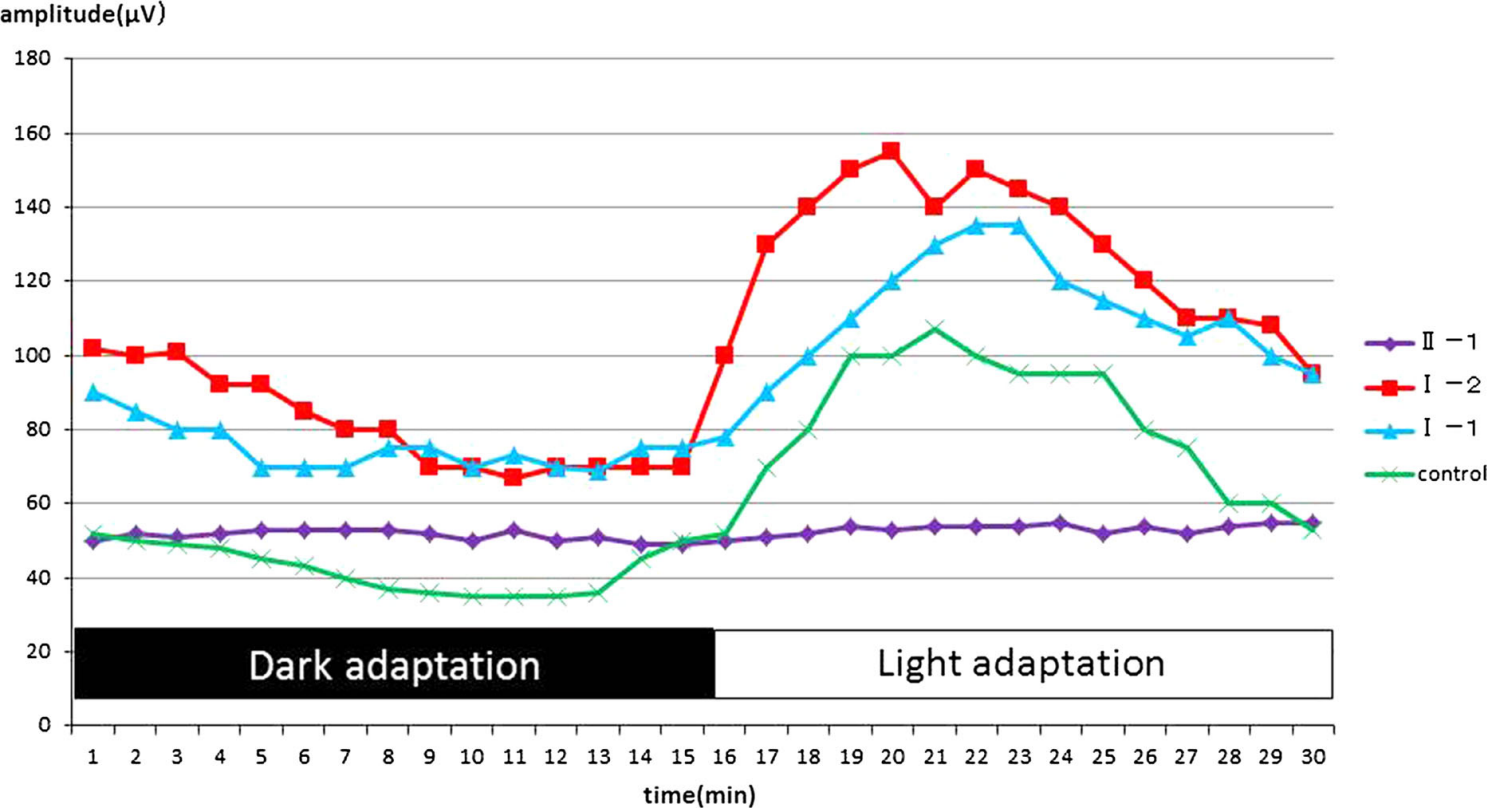
Electro-oculograms (EOGs). The EOGs of the right eyes of the proband, proband’s parents, and normal control are shown. The Arden ratio of the EOG of the proband is markedly reduced with an absence of the light rise. The EOGs of the parents have a normal Arden ratio and light rise

The amplitudes of both the cone and rod full-field ERGs were reduced, and the waveforms were similar in both eyes (Fig. 2). The amplitudes of the mfERGs were reduced in the central and peripheral sectors of both eyes (Fig. 3). The Arden ratio of the EOG was 1.1 in both eyes with a dark trough 15 min after beginning the measurements and a light peak 15 min from the beginning of the light phase (Fig. 4).

**Fig. 5:**
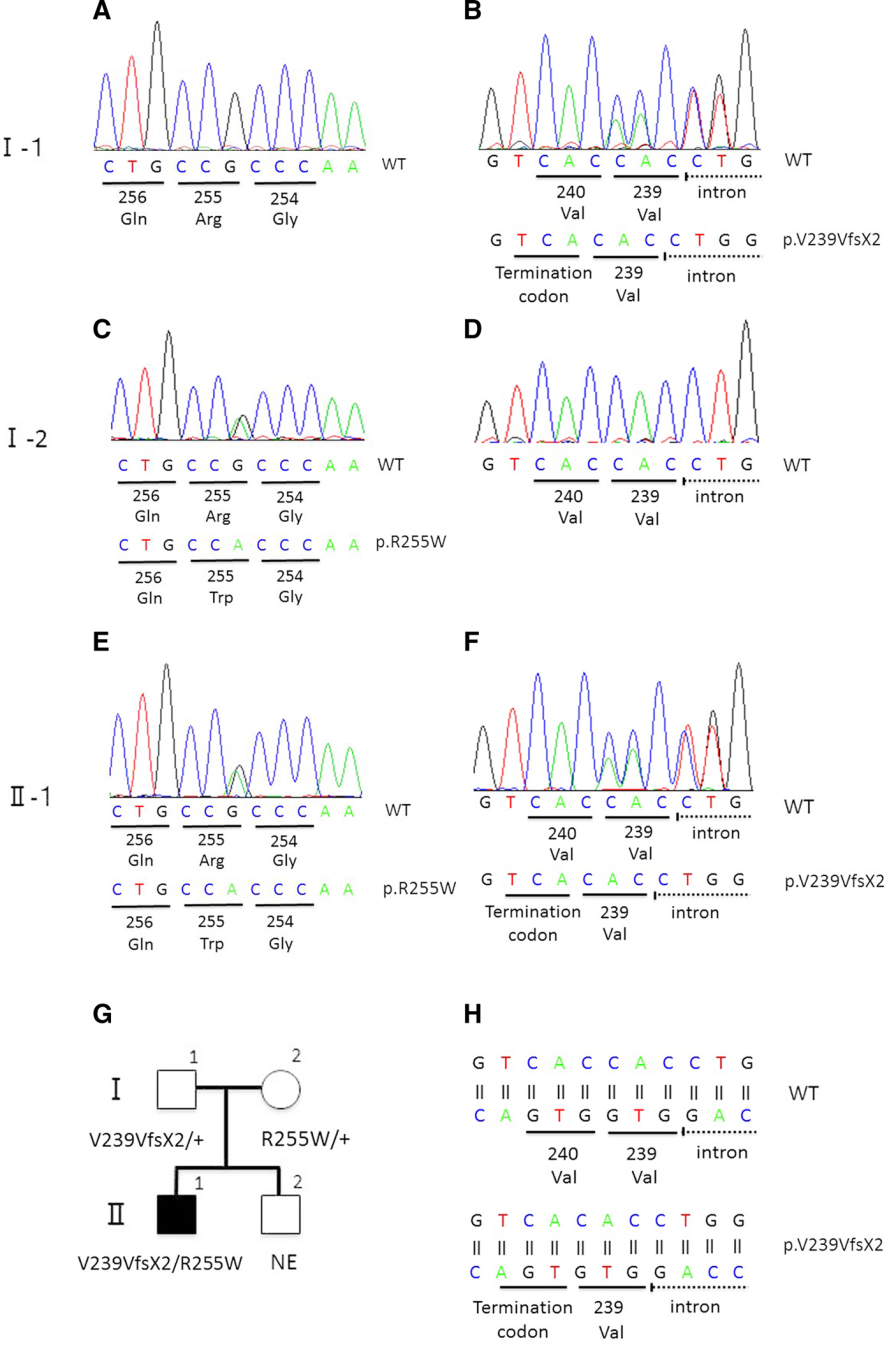
Molecular genetic findings and a pedigree chart. Sequence chromatograms of the proband’s father (I-1; **a**, **b**), mother (I-2; **c**, **d**), and the proband (II-1; **e**, **f**) are shown. Sequence chromatograms around the amino acid position 255 (**a**, **c**, **e**) and 240 (**b**, **d**, **e**) are shown. Results of reverse strand of the sequence chromatograms are shown (**a**–**f**). A single-nucleotide mutation (c.763C>T) results in the substitution of tryptophan for arginine at amino acid position 255 (p.R255W) in the mother and proband (**c**, **e**). A deletion mutation (c.717delG) results in the synonymous substitution of valine for valine at amino acid position 239 and a frame shift that leads to a premature termination codon at two amino acid residues downstream from the mutation (p.V239VfsX2) in the father and proband (**b**, **f**). Pedigree charts for the segregation analysis are shown (**g**). Schematic diagram of the deletion mutation (c.717delG) in the proband (*bottom*) and wild type (*top*) are shown (**h**). A frame shift mutation leads to a premature termination codon at two amino acid residues downstream from the mutation

Mutation analysis of the *BEST1* gene in the proband showed two heterozygous sequence variants. One was a novel variant, c.717delG, p.V239VfsX2, and the other was a variant previously reported, c.763C>T, p.R255W. Both variants were found in exon 7 (Fig. 5).

**Fig. 6:**
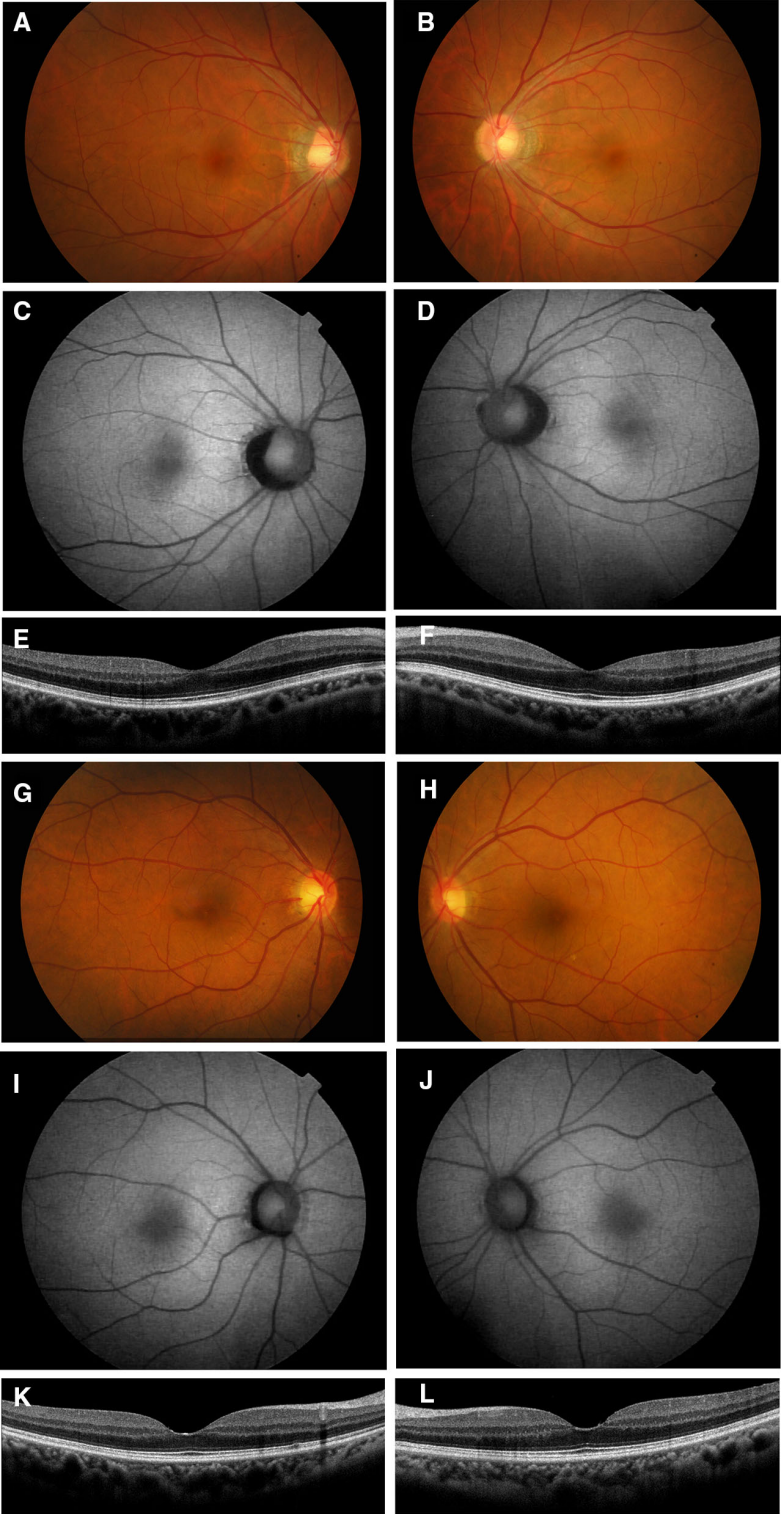
Fundus photographs, fundus autofluorescence image, and SD-OCT images from the parents of the proband. Fundus photographs (**a**, **b**, **g**, **h**), autofluorescence (**c**, **d**, **i**, **j**), and SD-OCT images (**e**, **f**, **k**, **l**) are shown. Results from the father (**a**–**f**) and mother (**g**–**l**) are shown. Fundus appearance, FAF, and SD-OCT of the proband’s parents are normal

The proband’s father (I-1, 57 years old) and mother (I-2, 57 years old) had normal visual acuity, and their fundus, FAF, and SD-OCT images were also within normal limits (Fig. 6). The EOGs of both parents had a normal light rise with normal Arden ratio in both eyes (Fig. 5). Mutation analyses of the parents identified a c.717delG, p.V239VfsX2 variant in the father and a c.763C>T, p.R255W variant in the mother in the heterozygous state.

## Discussion

The imaging and functional data obtained on our patient are in good agreement with the findings from previous reports of ARB. The characteristic features of ARB are a clinically recognizable retinal dystrophy with yellowish subretinal lesions scattered in the posterior pole that have marked diffuse fundus autofluorescence abnormalities [18–24]. The SD-OCT findings of previous ARB cases included diffuse intraretinal cystic spaces across both the inner and outer plexiform layers, subretinal fluid with shallow serous retinal detachment, and thickening and hyper-reflectivity of the ellipsoid and interdigitation zones which may represent an elongation of the photoreceptors [21, 24, 25]. The Arden ratio of the EOGs of patients with ARB is reported to be low with an absence of the light rise [11, 19, 23]. The imaging and functional findings in our patient are typical of ARB.

The *BEST1* mutation, c.717delG, p.V239VfsX2, has not been reported and not included in the SNP database. The allele frequency of the variant was estimated from two databases; the Human Genetic Variation Database (HGVD; http://www.genome.med.kyoto-u.ac.jp/SnpDB/about.htm) which is specific for the Japanese population, and the ExAC Browser (Beta)(http://exac.broadinstitute.org) database. Both of these databases did not contain the allele frequency of the variant, which indicates that this variant is very rare.

Although most mutations associated with BVMD are missense mutations that do not compromise protein synthesis, the few ARB-causing mutations reported to date are premature truncations or nonsense substitutions that lead to early transcript degradation or non-functional proteins. These are associated with a null phenotype (Table 1). In truncating *BEST1* mutations, the null phenotype associated with ARB is attributed to a severe decrease in *BEST1* expression promoted by the nonsense-mediated decay (NMD) surveillance mechanism [26]. Recent evidence supports the idea that NMD degradation depends on the position of the premature translation termination codons. Pomares et al. [26] reported that the *BEST1* transcripts in a patient who carried the premature stop codon at position 230 are preserved in only 13 % of the case, while the *BEST1* transcripts of a patient who carry a premature stop codon in position 349 are preserved in 22 % of the case. Patients who carry the premature stop codon in position 230 have a characteristic ARB phenotype, while patients who carry a premature stop codon in position 349 have ophthalmological features resembling both ARB and BVMD [26]. Thus, the residual amount of aberrant protein can promote a negative effect causing a mixed phenotype of both ARB and BVMD traits. This hypothesis was supported by previous reports of biallelic *BEST1* mutations with at least a premature termination codon (Table 1). Although patients 2 and 6 of Table 1 had the BVMD phenotype which is not consistent with the hypothesis, the same second allele mutation (R141H) may be associated with the BVMD phenotype [21, 27]. Our patient with a premature termination codon at position 240 is consistent with the hypothesis that the patient should have an ARB phenotype.

**Table 1:** Summary of previously reported biallelic *BEST1* mutations with premature termination codon

Patient number	Allele 1			Allele 2				
	Nucleotide	Amino acid	Termination position	Nucleotide	Amino acid	Termination position	Fundus appearance	References
1	c.15C>A	p.Y5X	5	c.430A>G	p.S144G		ARB	Lacassagne et al. [30]
2	c.87C>G	p.Y29X	29	c.422G>A	p.R141H		BVMD	Schatz et al. [27]
3	c.102C>T	p.E35WfsX11	45	c.1470_1471delCA	p.H490QfsX24	513	ARB	Davidson et al. [18]
4	c.172_173dupCA	p.Q58HfsX4	61	c.584C>T	p.A195V		ARB	Borman et al. [21]
5	c.175_176dupCA	p.Q59HfsX3	61	c.175_176dupCA	p.Q59HfsX3	61	ARB	Boon et al. [22]
6	c.475C>T	p.Q159X	159	c.422G>A	p.R141H		BVMD	Borman et al. [21]
7	c.519delA	p.K173NfsX2	174	c.860G>A	p.W287X	287	ARB	Tian et al. [29]
8	c.598C>T	p.R200X	200	c.598C>T	p.R200X	200	ARB	Burgess et al. [11]
9	c.263_279del17	p.L88LfsX138	225	c.584C>T	p.A195V		ARB	Gerth et al. [25]
10	c.521_522delTG	p.L174EfsX57	230	c.521_522delTG	p.L174EfsX57	230	ARB	Pomares et al. [26]
11	c.762delG	p.R255GfsX4	258	c.74G>A	p.R25Q		ARB	Boon et al. [22]
12	c.1100 + 1G>A	p.V317PfsX33	349	c.1100 + 1G>A	p.V317PfsX33	349	ARB + BVMD	Pomares et al. [26]
13	c.1066C>T	p.R356X	356	c.550C>T	p.P184S		ARB	Borman et al. [21]
14	c.1038duC	p.Y347LfsX54	400	c.553A>C	p.H178P		ARB	Borman et al. [21]
15	c.1212delC	p.P404PfsX78	481	c.637G>A	p.E213K		ARB	Silva et al. [31]
16	c.1415delT	p.L472PfsX10	481	c.1415delT	p.L472PfsX10	481	BVMD	Bitner et al. [16]
17	c.1470_1471delCA	p.H490QfsX24	513	c.584C>T	p.A195V		BVMD	Kinnick et al. [32]
18	c.1669delG	p.E557NfsX52	608	c.934G>A	p.D312N		BVMD	Sodi et al. [33]

The other mutation found in this study (R255W) was reported to be present in both a BVMD family in the heterozygous state and two ARB families in the compound heterozygous state [28, 29]. In the BVMD family with the R255W mutation, the parents of the proband were not genetically examined [28]. In the ARB families with the R255W mutation, each parent of the proband was heterozygous carriers of the R255W mutation and they were healthy [29]. Our data do not explain why the mother of our patient who carried the heterozygous R255W mutation did not have BVMD. One possibility is that the mutation exhibits reduced penetrance for the phenotype. The other possibility is that the previously described BVMD patient who had heterozygous c.763C>T, p.R255W mutation may have had an undiscovered second allele mutation such as a large deletion.

In some cases, it is difficult to differentiate ARB from BVMD and to speculate on the prognosis of the disease. Identifying the genetic defect of *BEST1* gene and position of the premature termination codon may help in differentiating the ARB from BVMD and predict the prognosis of the disease.
